# The Impact of Palliative Care on Mitigating Pain and Its Associated Effects in Determining Quality of Life among Colon Cancer Outpatients

**DOI:** 10.3390/healthcare11222954

**Published:** 2023-11-12

**Authors:** John M. Macharia, Bence L. Raposa, Dávid Sipos, Csaba Melczer, Zoltan Toth, Zsolt Káposztás

**Affiliations:** 1 Doctoral School of Health Sciences, Faculty of Health Science, University of Pẻcs, Vörösmarty Str 4, 7621 Pẻcs, Hungary; 2 Faculty of Health Sciences, University of Pécs, Vörösmarty Str 4, 7621 Pẻcs, Hungary; 3 Department of Medical Imaging, Faculty of Health Sciences, University of Pécs, Szent Imre Str 14/B, 7400 Kaposvár, Hungary; 4 Institute of Physiotherapy and Sport Science, Faculty of Health Sciences, University of Pécs, Vörösmarty Str 4, 7621 Pẻcs, Hungary; csaba.melczer@etk.pte.hu

**Keywords:** quality of life, palliative care, hospice, pain, colon cancer, outpatient

## Abstract

Pain continues to be a significant problem for cancer patients, and the impact of a population-based strategy on their experiences is not completely understood. Our study aimed to determine the impact of palliative care on mitigating pain and its associated effects in determining the quality of life (QoL) among colon cancer outpatients. Six collection databases were used to perform a structured systematic review of the available literature, considering all papers published between the year 2000 and February 2023. PRISMA guidelines were adopted in our study, and a total of 9792 papers were evaluated. However, only 126 articles met the inclusion criteria. A precise diagnosis of disruptive colorectal cancer (CRC) pain disorders among patients under palliative care is necessary to mitigate it and its associated effects, enhance health, promote life expectancy, increase therapeutic responsiveness, and decrease comorbidity complications. Physical activities, the use of validated pain assessment tools, remote outpatient education and monitoring, chemotherapeutic pain reduction strategies, music and massage therapies, and bridging social isolation gaps are essential in enhancing QoL. We recommend and place a strong emphasis on the adoption of online training/or coaching programs and the integration of formal and informal palliative care systems for maximum QoL benefits among CRC outpatients.

## 1. Introduction

Cancer is the second leading cause of death after cardiovascular disease [1]. Localized tumors with limited growth are classified as benign, whereas those that spread to other parts of the body and are aggressive on healthy tissues are classified as metastasized and malignant [1,2]. Colorectal cancer is the third most common cause of cancer-related mortality and morbidity in Western countries [3]. Due to a lack of alternative intervention options, chemotherapeutic therapies, radiotherapy, and surgery are the kinds of intervention that are most often used for colorectal tumors. As a result, therapy based on plant-derived dietary supplements is receiving increased attention as the most effective means of reducing the burden of colon cancer-related mortality [4]. The existing nonsurgical palliative therapies in colon and rectal cancer are focused on symptom alleviation, as well as pain relief. Historically, when patients presented with acute symptoms like blockage, they were treated with an emergency operation, usually requiring the insertion of a stoma [5].

Pain is “an unpleasant sensory and emotional experience associated with actual or potential tissue damage, or described in terms of such damage”, according to the International Association for the Study of Pain (IASP) [6]. A broad phrase used to describe a variety of pain disorders with various physiological properties is “cancer pain” [7]. Tumors themselves, oncological therapies (such as chemotherapy, radiotherapy, surgery, or immunotherapy), and tissue damage can all cause pain [7,8]. Pain is a frequent, complicated, and frustrating symptom for cancer patients. Importantly, pain can be physical, but it can also have psychosocial and spiritual elements since it is whatever the person who experiences it claims it is and occurs whenever they say it does [9]. Total pain is the term associated with this condition, which is adequately taken into account in palliative care [10]. According to reports, up to 80% of cancer patients experience distress that lowers their quality of life (QoL) in terms of overall well-being [11]. Moreover, Fallon et al. noted that, among cancer patients with an advanced disease, pain was found to affect more than 70% of patients [9]. Unfortunately, this could also have a substantial impact on ambulatory individuals who are still in the early stages of their illness [12]. It is currently unclear how chronic pain after colon cancer surgery develops. In particular, the lack of a validated technique for measuring chronic pain is a serious problem because it is essential for determining the incidence and risk factors [13]. The International Association for the Study of Pain (IASP) created a systematized taxonomy of chronic pain syndromes that distinguishes between chronic primary and chronic secondary pain syndromes. Chronic pain is defined as pain that lasts or recurs for more than three months. The term “chronic primary pain” is used in some circumstances where pain may be regarded as a sickness. However, in some instances, such as with chronic cancer-related pain, pain is a complication of an underlying illness [14].

It has been estimated that pain prevalence ranges from 33% in patients following curative treatment to 59% in patients receiving anticancer treatment and 64% in patients suffering from metastatic, advanced, or terminal disease [9]. In a study performed by Morita et al., they demonstrated that regional palliative care programs did not show any improvement in pain intensity among cancer outpatients. They postulated that a possible interpretation could be that they are less likely to be considered target populations [12]. Pain is still a significant issue for cancer patients, and the impact of a population-based strategy on their experiences is not completely understood [9,12].

Undertreatment is prevalent, despite guidelines and the availability of opioids (the basis of moderate-to-severe cancer pain management). European studies [15] corroborated these findings from the United States, revealing that various types of pain or pain disorders were present at all phases of cancer and were not appropriately managed in a large proportion of patients, spanning from 56% to 82.3%. According to a 2014 systematic review [16] that used the Pain Management Index (PMI) [17], about one-third of patients do not obtain analgesia commensurate to their pain level. Palliative care has deservedly received more attention in recent years. Palliative therapy is usually explored by surgeons and oncologists when excision of the tumor is no longer possible. Providing the best palliative care for a patient with advanced colorectal cancer is a complicated task. The process of providing palliative care may differ from the typical surgical gratification received from the complete excision of cancer, but surgeons who achieve excellence in palliative care will most certainly find it fulfilling [5]. Physical pain management, symptom control, information exchange, advance care planning, spiritual and emotional support, and care coordination are the main issues associated with pain and relevant to palliative care. Through a wholistic approach, palliative care strives to improve QoL for those with terminal illnesses and their families [5,18].

It is critical to launch global campaigns to increase awareness, knowledge, and expertise of this topic among oncologist specialists treating and managing cancer patients and caregivers to decrease suffering and improve QoL among CRC outpatients. Pain, being a symptom experienced by more than 70% of colorectal cancer (CRC) patients, is still a dreaded and severe side effect of both cancer and cancer-associated treatment [19]. It is against this backdrop that the current study aimed to determine the impact of palliative care on mitigating pain and its associated effects in determining the quality of life (QoL) among colon cancer outpatients. There is sufficient proof adduced in this updated review that palliative care can lessen physical discomfort and agony, lessen psychological and spiritual anguish, cut down on unneeded hospital admissions and length of stays, and increase survival rates among CRC outpatients, thus promoting a healthy QoL (H-QoL).

## 2. Methods

### 2.1. Electronic Database

**Figure 1:**
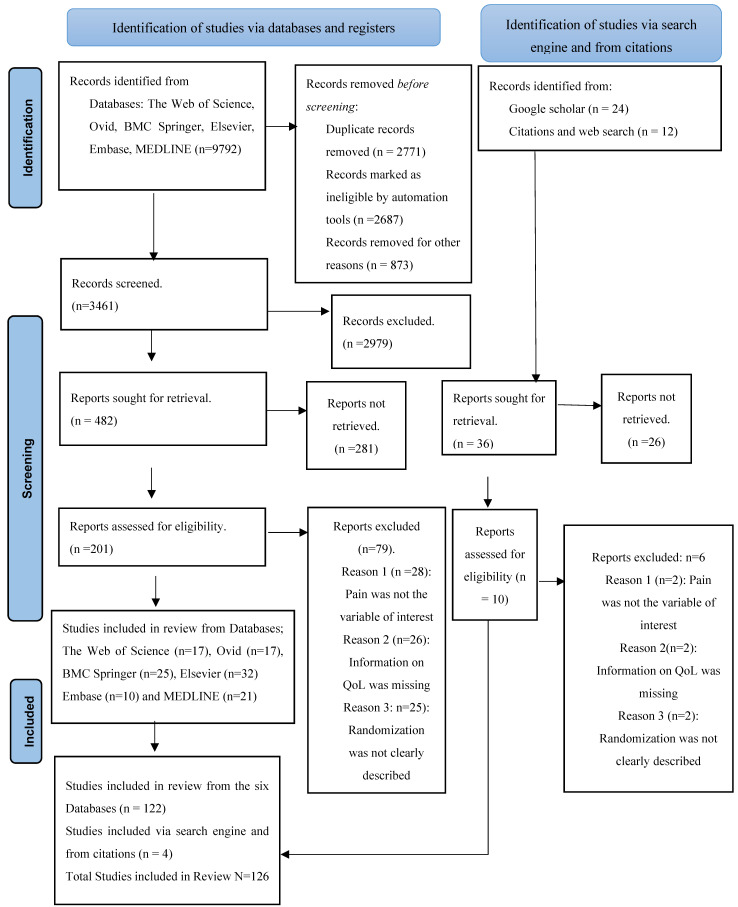
Results were yielded using an adopted PRISMA flow diagram with minimal modifications for the conducted systematic review.

For screening and selection, a thorough search of the relevant literature was conducted using the Web of Science, Ovid, BMC Springer, Elsevier, Embase, and MEDLINE databases in full adherence to already established PRISMA guidelines [20], but with slight modifications (Figure 1). In addition, Google Scholar was used to supplement material from additional articles pertinent to our study. Importantly, before being used in this review, papers from Google Scholar were evaluated for credibility and authenticity [21] by correlating them with the relevant publication, considering its true publisher [22,23] and registered identifiers.

### 2.2. The Screening Criterion and Search Strategy

The authors began by independently screening only English-language publication titles and abstracts from primary investigations, considering all articles published till November 2022. The exclusion of non-English language publications was necessitated by the lack of professional expert interpretation of potential articles. However, we expanded the search term strategy to obtain significant publications to support a thorough study of acceptable standards globally. Studies on malignancies other than CRC, as well as research on in-patients, were excluded from the study. Titles and abstracts that satisfied the established criteria were recruited for full-text article evaluation and afterward used to provide the necessary analytical data for the current review after being deemed appropriate by three independent investigators. The author’s independence was maintained when determining whether or not to incorporate the enrolled articles to reduce the potential risk of bias [21]. The data collection forms were standardized to remove discrepancies that could potentially arise and allow the three reviewers to get the same data from each study. A technique for dispute settlement was previously suggested, much like the procedure for study selection.

The quality/bias of included research was determined by, among other things, whether the included studies had minimal bias in their study design (internal validity), as has been suggested and performed in other studies [24]. Nonetheless, the following factors were used to determine the quality of each study: (1) research characteristics, such as author, publication year, and country; (2) sample characteristics, including sex, age, type of palliative care, intervention, protocol, and related outcome; (3) primary and secondary outcomes, including indicators of pain and pain relief, bowel function, activities of daily living (ADL), and QoL; and (4) duplicate studies and those with overlapping participant and study period ranges were excluded from the group of studies published by the same research team.

Key search words and associated combinations were systematically utilized across all the databases looked up [10], as was performed by other investigators in similar studies.

Key terms: “palliative care”, “hospice”, and “home care” terms were combined with either of the following MeSH terms: “pain”, “physical pain”, “psychosocial”, “emotional”, “spiritual need”, “agony”, “stress”, “depression”, and “fear”, using the Boolean operator AND. These keywords were only used in conjunction with one of the following names: “colorectal tumor”, “colorectal cancer”, or “colon cancer”, using OR as the appropriate Boolean operator to effectively yield sufficient results.

#### Applied Ovid Search Strategy

To retrieve the most relevant search results from the database, appropriate strings were constructed, and the “advanced (syntax) search” option was duly activated. In addition, besides other journal search options, Ovid Medline database, a significant component in the Ovid database was considered too. The Ovid database was utilized to map our keyword phrases to MeSH for an efficient search of matching content rather than text. Finally, “explode” and “focus” options were checked to reflect the major points in the searched article and for a more defined search approach. A typical search string used in our research study was combined as follows: “palliative care” OR “hospice” AND “pain” OR “physical pain” AND “colorectal cancer” OR “colon cancer”. This search strategy was repeated using different keywords and MeSH terms determined for application in this study to screen for more results.

## 3. Results

### 3.1. Successfully Enrolled Full-Text Articles

**Figure 2:**
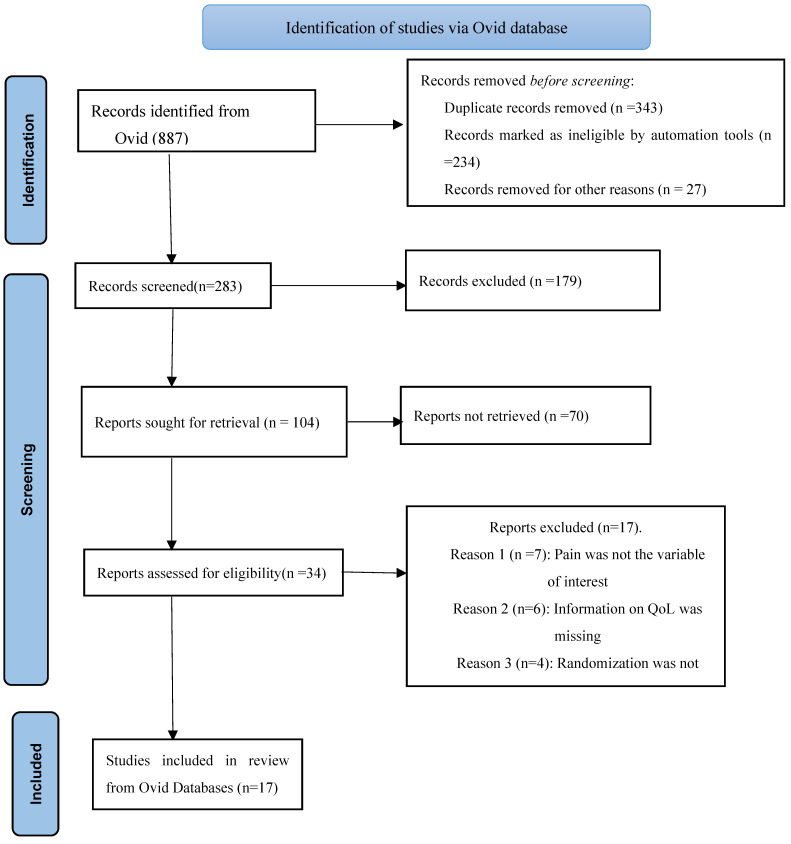
A simplified schematic representation of all studies retrieved from the Ovid database for inclusion in the study.

An appropriate pain evaluation is the first step in effective pain therapy, according to the American Pain Society and the European Task Force on Cancer Pain [25]. Pain is a common and debilitating cancer symptom, disrupting patients’ lives sometimes even more than the cancer itself [26]. This review unveils that that screening for pain in cancer outpatients improves the quality of care and, more importantly, pain-related results [27]. A total of 9792 publications that were published between the years 2000 and December 2022 were found through the entire screening process. However, a total of 9684 publications were eliminated from the study because they did not match the inclusion criteria. In total, 126 articles satisfied the inclusion requirements and were adequately explored in this updated and comprehensive systematic review, in compliance with the appropriate PRISMA guidelines [20] mentioned earlier. From the Ovid database, only 17 full-text articles were successfully retrieved for inclusion in the study (Figure 2).

### 3.2. Pain: The Impact of Pain as a Factor Influencing QoL among CRC Outpatients

The histologic type of the cancer, the location of the main tumor, and the location of metastases are the three factors that determine the clinical presentation of cancer pain [26]. Cancer pain can be controlled using several methods. There are currently pharmacological and nonpharmacological therapies available, but they are not always effective, and many patients continue to suffer from pain [15]. The consequences of untreated or inadequately treated pain can be severe, impacting physical health, psychological health, and interpersonal relationships [28]. Persistent pain has a severe impact on the QoL of cancer patients: as a response, individuals may fear pain more than death from the cancer, and this anxiety has strengthened the push for physician-assisted suicide [29]. In recent years, significant advances in the management of cancer pain have included better pain assessment, detection, and treatment of opioid-induced neurotoxicity, as well as the rising use of opioid rotation and methadone [15].

Cancer pain is, nonetheless, pervasive and incapacitating, notwithstanding the evidence–practice gap that prevails in this area [30]. Effective cancer pain assessment and management necessitates patient self-reporting using patient-reported outcome measures (PROMs) and compliance to management strategy; health professionals with the commitment, knowledge, and skills to manage pain; and health services that provide a screening method to identify and monitor symptoms [27,31]. It has been shown that testing for pain in cancer patients receiving outpatient care enhances both the standard of care and, more crucially, outcomes linked to pain [27]. Of significance, pain is a subjective experience that is best evaluated from the patient’s point of view, which is easily performed with standardized PROMs [31]. A handbook created by the International Society for QoL Research offers helpful guidance for clinicians and researchers wishing to integrate PRO assessment into common clinical practice [32].

#### 3.2.1. Colorectal Cancer-Derived Pain (Physiological Effects)

CRC-derived pain is still a dreaded and unpleasant physiological side effect of both cancer and cancer treatment. In CRC, there are numerous pain management choices, including intravenous, oral, and topical medicines. Proper treatment should target the nociceptive, neuropathic, and/or psychogenic pain elements to cover the whole continuum of pain [19]. Neuropathic, somatic, and visceral pain are the three basic forms of pain. The latter two are included in the category of nociceptive pain, which is pain brought on by bodily tissue injury. However, neuropathic pain is connected to nerve injury [33].

Despite being a challenging clinical issue and a complex pathologic process, understanding the fundamental neurologic mechanisms behind CRC pain has advanced significantly. The cellular, tissue, and systemic modifications that take place during proliferation, invasion, and metastasis are the cause of the symptoms that these CRC patients feel. The primary afferent nociceptor and the cancer collaborate and communicate dynamically during nociception [26]. Surprisingly, endothelin-1’s (ET-1) effects on cancer pain are intricate. Two endothelin receptor subtypes that variably influence opioid release from carcinomas are essential for understanding these effects. A powerful vasoactive peptide called ET-1 causes nociceptive behavior in both humans and animals [34] and is the primary cause of CRC-derived pain [34]. Even though several types of cancer produce ET-1, not all tumors do [35,36]. Inflammatory pain, which is frequently brought on by the compression of nerve terminals brought on by the growth of tumors, can be caused by the production of inflammatory chemicals and sensitization of nociceptors [37].

Currently, treatment options do not provide pain relief to a substantial number of individuals and, when used incorrectly, can lead to a variety of complications [19]. The development of a superior tool for measuring undertreatment, educational interventions to enhance healthcare workers’ pain management skills, and the creation of more effective and individualized pharmacological and nonpharmacological pain treatments are all recommendations that are strongly advised to improve the treatment of CRC-derived pain and all other cancer types by extension [27]. However, it has not demonstrated that the type of cancer is a predictor of the presence of pain, even though patients with gastrointestinal, lung, breast, other hematological, and “other” malignancies have a substantially higher risk of experiencing moderate to severe pain than those with prostate cancer [38]. As earlier stated, opioid-based pharmacotherapy is frequently used to treat cancer pain; however, these medications have several side effects. Transient receptor potential channels (TRPs) are one novel method being studied for treating cancer pain. TRP ion channels, particularly TRP vanilloid 1 (TRPV1) and TRP ankyrin 1, are expressed in many tissues and are important for the detection of pain (TRPA1). TRP channels are thus prospective targets for treating pain disorders associated with malignancy [37].

The memory of cancer-derived pain frequently contains errors and is affected by a variety of contextual circumstances [39]. Patients typically underestimate their discomfort when they have cancer since it is thought to be directly tied to the progression of the illness [40]. Contrary, since pain serves as an alarm bell that prompts the body to take action to protect itself, this could worsen the subject’s H-QoL [41]. It is also possible for medical practitioners to neglect performing normal pain assessments since they are more focused on diagnosing and treating cancer [33]. In this case, the evaluation is seldom performed, typically in clinical settings. Some CRC patients may already be struggling with substance abuse. The prevalence is comparable to that of the general population, and many of the patients are being looked after by relatives who have a history of drug abuse, addiction, or misuse [42]. Assessing and managing pain in such persons with substance-use disorders might be more challenging [43].

The deployment of a wearable gadget for ubiquitous recording in a real-world setting; the implementation of a big-data strategy that may be aided and abetted by artificial intelligence and machine learning, including multiple stratification factors (e.g., cancer location and phase, the origin of pain, and demographic and psychosocial data); and effectively recording procedures are all factors that need to be performed to obtain a reliable method for assessing CRC-pain among outpatients receiving palliative care. The management of cancer pain among patients could then benefit greatly from improved methodologies and algorithms [7]. Strong and reliable pain measurement tools are those that have consistent backup research from several authors. The use of trustworthy and dependable instruments helps to guarantee that physicians are applying the right standards in their pain assessments [44]. Using standardized tools encourages consistency among healthcare professionals and makes it easier to communicate and assess pain management options for treatment [7,44]. Table 1 represents simple validated pain assessment tools.

Clinicians face a hurdle when assessing pain in cognitively impaired cancer patients. The pain management care plan would be simplified by a pain assessment instrument that can detect the presence of pain or a decline in pain behaviors. However, the absence of pain behaviors does not imply that the patient is pain-free. Although tools based on nonverbal pain behaviors have been developed to check for and assess pain in this vulnerable population, none of them is recommended for use in oncology settings [33,43].

The Multidimensional Objective Pain Assessment Tool (MOPAT) was recently created to evaluate acute pain in patients who are unable to self-report in palliative care settings [45]. It has two acute pain dimensions that are intended to examine behavioral dimension markers (restlessness, muscle tension, facial expression, and vocalizations), as well as physiologic dimension markers (blood pressure, heart rate, respiratory rate, and diaphoresis). It is the only tool with proof of its validity, dependability, and clinical significance in palliative care environments [45,46]. Only a small number of tools have been created and approved specifically for use with people nearing the end of their lives. While vital indicators (e.g., changes in heart rate, blood pressure, and respiratory rate) may be useful for identifying the deleterious consequences of severe pain, they are not reliable for distinguishing pain from other causes of discomfort [47]. Disruptions in vital signs may not always signal pain, and the absence of fluctuation in vital signs does not always imply the absence of pain. Vital signs should only be used as signals to initiate additional evaluation, using approved and validated methods such as the patient’s self-report of pain (where possible) or a behavioral pain assessment instrument suggested in our discussion [48].

#### 3.2.2. CRC-Associated Pain and Their Possible Palliative Strategies

##### Fear, Anxiety, and Depression (Psychological Effects)

Pain from colon cancer is harmful to the patient’s psychological health. Reduced adherence to therapy results from the lower QoL, which inevitably has negative effects. Cancer discomfort has a financial cost in addition to physical and societal costs [49,50]. Anxiety is a typical reaction to receiving a cancer diagnosis and a reasonable reaction to perceived dangers like losing body functions, changing one’s appearance, upsetting one’s family, dying, etc. In cancer patients, anxiety frequently coexists with depression and may last throughout the course of the disease, greatly lowering the patient’s quality of life [51].

It has been suggested that cancer outpatients receiving care are adversely confronted with the fear of cancer recurrence (FCR), which significantly influences their QoL [52]. A high degree of attention, high levels of anxiety, persistence, and hypervigilance to visible symptoms are hallmarks of clinical FCR [53,54]. Although feeling apprehensive in the face of risks like cancer is normal, some patients experience extreme anxiety that makes it difficult for them to go about their daily lives. Anxiety frequently rises as the illness worsens or as the course of treatment becomes harsher [55], as well as at transitional periods that symbolize dangerous occurrences along the course of the illness. After initial shock or disbelief, patients who receive a cancer diagnosis, learn of a recurrence, or realize that therapy has been ineffective frequently endure emotional upheaval, anxiety, and depressive symptoms [51]. It has been established that anxiety can influence how someone behaves concerning his/her health, which can lead to a delay in or omission of actions that could effectively prevent or treat cancer. In addition, it might cause one to overestimate the likelihood of a bad outcome [51,56]. Since the intervals between cancer recurrences are strongly correlated with mortality risk, cancer researchers place a high priority on preventing cancer recurrence [57]. Nonsteroidal anti-inflammatory medicines (NSAIDs) may be able to delay the development of cancer and increase patients’ five-year survival rates, according to some research [58]. The survival rate of those with colorectal adenocarcinoma is also improved by metformin [59]. These findings imply that cancer chemoprevention, an efficient method for raising patients’ five-year survival rates, may be beneficial for those who are at higher risk of cancer incidence and recurrence.

Post-treatment depressed-symptom reduction has been linked to both music and massage therapies; however, music therapy is more strongly linked to symptom reduction than massage therapy [60]. While massage therapy has a relaxing impact, music therapy has an invigorating effect. Previous studies have shown that the monoaminergic systems that antidepressants target are modulated by music and massage [61]. Variations in reward, motivation, and arousal-related brain regions could potentially contribute to the invigorating benefits of music therapy; changes in the activity of these brain regions have been seen after joyful musical experiences [62]. Possible explanations for the calming benefits of massage therapy include an induced switch from sympathetic to parasympathetic activity in response to touch [63]. The comparative effectiveness of music therapy and massage therapy for depressive symptoms among CRC outpatients, however, requires randomized clinical research for strong validation.

Worth significant attention, as reported earlier and supported by recent studies, is the fact that one of the most prevalent unmet needs among cancer patients is FCR or progression [54]. To evaluate FCR, many verified metrics/tools have been created [64]. The nine-item Fear of Cancer Recurrence Inventory-Short Form (FCRI-SF) is one of the most frequently used and validated tools in this field [65]. Given its length, the more recently created four- and seven-item Fear of Cancer Recurrence scale (FCR4/7) may be perfectly applied for medical evaluation [66]; it examines clinical FCR features. Besides evaluating other effects, it is critical to address this detrimental physiological effect among CRC outpatients under hospice care for promising QoL outcomes, using validated or improved clinical psychometric tools. However, to empower researchers and clinicians to effectively address this demand among patients from low- and middle-developing economies across the cancer care continuum, studies are required to translate and culturally validate FCR metrics/tools.

##### Sleep Disorders among CRC Patients and Mitigation Approaches

Sleep disorders are a typical sign of worry, one of the top concerns for cancer patients, and a major driver of oncology consultations [67]. People with insomnia generally report greater medical issues than people without insomnia [68]. Altering sleep typically has a severe negative impact on one’s ability to function emotionally, cognitively, and physically. Along with the physical sickness itself, pain, hospitalization, and specialized medical therapies, sleep difficulties are usually linked to the psychological effects of cancer [51]. Changed sleep negatively impacts emotional health and performance during the day and, in the context of oncology, may be a precursor to delirium. Chronic sleeplessness has been linked to an increased likelihood of acquiring clinical anxiety or depression in the general population [69]. In addition to the physical sickness itself, pain, hospitalization, and specialized medical treatments, sleep disorders are strongly associated with the psychological effects of cancer [51,69].

People with chronic conditions such as CRC may experience insomnia, which could increase their symptoms, drastically lower their QoL, and possibly interfere with therapy [70,71]. For example, some studies suggest that sleeplessness lowers pain thresholds, and pain is the leading predictor of impairment [72]. Additionally, there is proof that sleeplessness is linked to lowered immunological function, which increases the risk of colds and flu in persons with cancer and may worsen their health [68]. Even during chemotherapy, most sleep disturbances in cancer patients are linked to inflammation: cytokines activate microglia (through humoral and/or neurological pathways), which can then lead to an astrocyte neurotoxic reaction. Adenosine, prostaglandins, nitric oxide, and GABA are just a few of the neurotransmitters that are affected by interleukin-1 beta (IL-1), which is produced in high amounts by tumors and inhibits REM sleep, while promoting non-REM sleep and inhibiting interleukin-6 (IL-6), which is produced in high quantities and appears to reduce REM sleep and increase slow-wave sleep [73,74].

The strong relationship between sleep and tumors is also influenced by hormones. For example, ghrelin, which has been linked to increased tumor growth and poorer survival, may play a role in orexin neuron activation [74]. Leptin, which is produced by, among other tumors, colorectal cancer, is involved in the increase in cancer cell proliferation and can induce the production of IL-6 and TNF-α, and it also seems to activate hypothalamic neurons, which are, in turn, connected with orexin neurons. Hormones are also involved in the close relationship between sleep and tumors [73]. Finally, a decrease in pH, hypoglycemia, and some amino acids can activate orexin neurons. Conversely, neurons that express the calcitonin gene-related peptide are sensitive to changes in pCO2 and are involved in the onset of arousals and awakening, whereas catecholamine activation, angiogenesis, an increase in tumor volume, and tumor invasiveness change the arousal response mechanism with the resultant sleep disruption/disorder [73,74].

Complex phenomena like the circadian rhythm, sleep, and cancer exhibit a dynamic interaction due to the regulation and moderation of overlapping molecular, physiological, and psychological processes [75]. Although the clock regulates gene expression, cell division, and DNA repair, the idea that clock genes are universal tumor suppressors has not been established [76]. Furthermore, carcinogenesis will undoubtedly lead to clock disruptions that impact biological activities by causing sleep deprivation and inadequate nutrient intake. Because of this Janus effect, there is a lot of interest in generating new medicinal approaches that manipulate circadian rhythms [75,76]. The mechanisms of coregulation are still poorly understood; however, these processes present an opportunity to improve palliative care for CRC outpatients. Although there is a symbiotic relationship between sleep and cancer, as demonstrated in our review, it is still unclear how certain sleep disturbances relate to human malignancies [74]. The improvement of sleep disorders may need the adoption of a sleep hygiene regimen. An ideal sleep hygiene regimen may vary depending on the patient. Sometimes the strategy calls for testing, dietary changes, medication adjustments, or relaxation-promoting methods and therapies [77]. If unwinding seems to be difficult for a patient, his/her caregivers may introduce him/her to several relaxation practices that might be beneficial. They are sometimes referred to as cognitive behavioral therapies. These techniques may include breathing exercises, constructive meditation, mindfulness, music, hypnosis, or guided imagery that assists people in unwinding physically and mentally. These practices assist patients in learning techniques for unwinding, remaining composed, falling asleep, staying asleep, and returning to sleep if they wake up during the night [77]. The correlation between exercise and sleep has been widely investigated and may be beneficial for improving sleep among CRC outpatients under palliative care [78,79]. To improve CRC outpatients’ health, an accurate analysis and understanding of their sleep disruption is required to alleviate pain.

##### Social Isolation and Loneliness and Intervention Approaches

Although it plays a big role in defining QoL, the relationships between loneliness, social isolation, and cancer are poorly understood. This notwithstanding, it has been reported that total cancer incidence is directly linked to both loneliness and social isolation [80]. While loneliness is the unfavorable perception of social isolation, that is, the subjective experience of being alone, social isolation refers to the objective lack of social relations with other people. According to some assertions, loneliness and social isolation have a negative impact on physical health just as much as some known health hazards, like smoking or obesity [81]. Previous studies have shown that living alone, particularly for men, shortens the length of time patients live after receiving a cancer diagnosis [82].

An important study elucidates that, social assistance may help break the relationship between loneliness and sadness [83]. By upregulating the hypothalamic–pituitary–adrenal (HPA) axis and autonomic nervous system, psychosocial stress affects the endocrine and immunological systems and can change a variety of physiological processes implicated in tumor formation [84]. Loneliness may be a significant psychosocial component linked to the severity of cancer [85]. The patient’s ability to begin therapy following the recommended treatment regimen, whether as an inpatient or an outpatient, depends on his/her ability to receive social support after obtaining a cancer diagnosis [86]. Further research is needed to clarify the mechanisms behind these relationships, which remain a mystery. Based on research findings, focused interventions for CRC outpatients who experience loneliness should be developed, regardless of gender, and they should be connected to available social networks while under hospice monitoring.

##### Improved Eating Habits and Physical Exercises

An increasing body of research also shows that certain cancer survivors may live longer, have a lower risk of the disease returning (or developing new cancer), and experience fewer side effects from therapy when certain dietary, physical activity, and associated factors are considered. Along with enhancing their general health and well-being, it can also reduce their risk of contracting some other serious diseases [21]. As a secondary prevention strategy, it has therefore been demonstrated that adopting a healthy diet and an active lifestyle can lower mortality from chronic disease. Fully explored and potent medicinal plants should also be considered important dietary supplements. The oxidation of lipids, proteins, and nucleic acids is inhibited by natural antioxidants present in medicinal plants, such as polyphenols and carotenoids, which stop the start of oxidative chain reactions. By scavenging unstable chemicals that could trigger carcinogenesis, these bioactive substances should be regarded as a crucial dietary supplement that could dramatically lessen the risks and incidences of colon cancer [21,87,88,89]. For example, sulforaphane, a compound found in cruciferous vegetables like broccoli, cabbage, cauliflower, and Brussels sprouts, has considerable anticancer therapeutic potential. Due to the biomolecule’s genesis in the seed rather than the growing broccoli plant, sulforaphane is found in the highest concentrations in broccoli sprouts. Mushrooms are also considered an anticancer dietary supplement [21,87,89]. Along with increasing sleep, bone health, and health-related QoL, physical activity can help lessen pain and its associated factors, like anxiety, sadness, fatigue, and lymphedema, among cancer survivors receiving palliative care [21]. Healthy nutrition, treatment compliance, and frequent exercise practices can all assist CRC survivors in improving their lifestyles [90].

##### The Side Effects of Colon Cancer Treatment and Preventive Approaches

The major methods for preventing cancer recurrence at present are chemotherapy or radiotherapy, but their usage is restricted due to their negative side effects and reported ineffectiveness [57]. Distress and emotional pain among CRC patients receiving palliative care might be brought on by therapeutic side effects. Anemia, cancer-related fatigue (CRF), exhaustion, weakness, hair loss, nausea, diarrhea, pain in the nerves, and mouth sores are side effects of chemotherapy [57,91]. It is critical to offer maximum care and emotional support to patients during seasons of these traumatizing experiences, especially hair loss! CRF is a prolonged, unpleasant, and subjective experience of exhaustion and/or drowsiness linked with cancer and therapy, encompassing physical, emotional, and cognitive components, and it can last more than five years after cancer treatment is completed [92]. When compared to a healthy person’s fatigue, CRF is more intense, more distressing, and much less likely to be improved by rest. CRF is considered to affect 25% to 99% of patients, depending on the patient population, type of medication administered, and technique of assessment [93]. Furthermore, concerning other negative clinical manifestations in cancer patients, such as sadness and anxiety, CRF has a more negative impact on QOL. As a result, intervention to alleviate CRF is prioritized in enhancing QoL following treatment completion [94].

According to recently updated reports, methods that target metabolism can stop the growth of cancer [95]. The formation of tumors is aided by metabolic reprogramming, which also creates metabolic liabilities that can be used to treat cancer. Chemotherapies that target metabolism have been successful cancer treatments for decades, and their efficacy shows that there is a pharmacological window to target malignant processes. These metabolic traits offer valuable targets for clinical use, as well as prospective cancer chemoprevention methods [59,95]. Since the glycolytic pathway supplies malignant cells with not just ATP but also biosynthetic intermediates for rapid growth and proliferation, lactic acid has been directly connected to the huge multiplication of cancerous cells [96]. The *LDHA*, *LDHB*, *LDHL*, and *hicD* genes are involved in this pathway and exchange metabolic fuel with the tumor stroma, making them promising targets for CRC chemotherapeutic drugs [96]. Greater emphasis should therefore be placed on the development of secure and much more effective LDH inhibitors.

### 3.3. Drawbacks and Adoption of Appropriate Follow-Up Programs for CRC Outpatients

Insufficient information, poor pain assessment, worries about rules and regulations, and fear of management implications (such as addiction, side effects, and tolerance) are the drawbacks/challenges that healthcare providers most frequently describe facing [97]. Effective hospice care settings/homes are frequently reported to face several obstacles, including healthcare systems that place little emphasis on pain management, expensive medication costs, issues with analgesic availability and accessibility, restrictive rules and regulations, and a lack of guidance and specialized support [97,98]. Remote monitoring and education programs can help cancer patients manage their pain more effectively, overcome obstacles, and boost treatment adherence, all of which will enhance their H-QoL [99]. Studies have demonstrated that effective pharmacologic pain management significantly reduces and improves pain in the majority of cancer outpatients [98]. In outpatient settings, nurses are strongly encouraged to remain vigilant and proactive by launching initiatives to improve patient adherence to the care plan and promote the necessity and appropriate management of cancer-related pain [100]. The initial and continuous assessment of patients experiencing cancer-related pain is carried out by nurses, who are at the forefront of providing treatment for these patients. A competent palliative care nurse’s duties should include creating a treatment plan, observing results, and educating patients and their families [100,101]. In addition to traditional palliative care, standardized pain education and monitoring administered remotely via phone calls by specialized nurses are effective in enhancing QoL. Therefore, offering remote outpatient education and monitoring to CRC patients could be a successful therapeutic strategy and should be highly encouraged.

### 3.4. Effectiveness of Online Support Programs for CRC Outpatients

The proactive use of information and communications technology (ICT) in the treatment of cancer patients is becoming more common [102]. The benefits of e-health-based self-management, web-based symptom management strategies, and mobile phone apps for cancer-related information demands have been supported by numerous research studies [103]. Many cancer patients struggle with how to manage their health and life since their real life is full of situations that undermine their health objectives, standards, and reference values. Yet, according to self-regulation theory, if one is sufficiently conscious of a disparity between current aspirations and reality, one can engage in a range of self-regulatory acts to lessen that difference [102]. Internet assistance programs and applications, such as a monitoring app, a confirmation app, and a writing app, have been created. These apps offer opportunities for cancer patients to monitor their health conditions (monitoring app), evaluate their understanding of the disease and its treatment (confirmation app), and address mental health issues to encourage cancer patients to identify and set their own mental well-being goals and modify their behaviors to achieve those goals (writing app) [104,105,106]. By using these apps, CRC outpatients may become more cognizant of the gaps between their reality, their goals, their expectations, and points of reference about their health and condition. Palliative care attendants should thus be equipped with such beneficial online tools to help improve the knowledge base of their CRC outpatients.

#### 3.4.1. Interactivity between Healthcare Providers, Patients, and Caregivers

The significance of e-health-based approaches can be expanded and tailored to support professional healthcare providers, patients, and caregivers within the same platform or space for maximum care benefits. Because of its unique advantages of ease of access and lack of geographic or time constraints, the Web-based format is a promising platform for delivering intervention to both healthcare providers and the caregivers of cancer patients [107]. Patient connection encompasses communication, physical contact, and emotional support, and its importance in healthcare cannot be overstated. The provision of information suited to the requirements of patients and carers is one part of optimum communication. Receiving more personalized and, thus, more personally relevant information has been linked to fewer unmet information needs [108], as well as improved psychological outcomes, such as lower anxiety levels [109]. Tailored information is not always provided, and this partly because healthcare practitioners may not know exactly what information a specific patient requires [109]. Patients and caregivers can express their information requirements by actively participating in medical consultations [110,111]. Many patients, however, report that they do not reach their ideal level of active participation during physical consultations [112]. To bridge these gaps between professional healthcare providers, patients, and caregivers, an enhanced tailored digital space could be a viable alternative that allows for the transmission of intended information in real time from either direction.

Online platforms are proving effective in sensitive cases, most especially where patients are required to freely open up without judgment, embarrassment, or social anxiety feelings. More than 80% of respondents in a Healthline-commissioned poll agreed that online cancer support groups and forums had a good impact on their cancer experience, particularly in terms of offering emotional support and making knowledgeable treatment decisions. Overall, social networking has evolved into a crucial tool for cancer patients and their loved ones to empower themselves and raise each other up [113]. Web-based interventions, such as online support groups alone or informational websites combined with online support groups, have been shown to significantly improve coping skills, as well as to lessen anxiety, stress, depression, burden, and negative moods in cancer caregivers. Indeed, research has shown that effectively managed interactions between healthcare providers, caregivers, and patients can improve a patient’s QoL by providing comfort and enjoyment [107,112,114].

However, as with any other kind of technology, there are risks associated with online platforms. They can, for example, cause information overload and provide dangerous or inaccurate advice. They can also reinforce some unhealthy behaviors. Furthermore, users may not be concerned about their privacy and confidentiality. Because of these dangers, it is critical to exercise extreme caution when browsing the broad web of online patient communities. As a result, caregivers can propose reliable sources of knowledge and verified and registered support groups. Patients can then broaden their social network by searching for and following people and organizations with similar interests. They must investigate the motivations of the groups that they are considering joining, as well as the relevance and usefulness of the information being exchanged with them [113,114]. Lack of Internet or unaffordable Internet services could potentially pose a major challenge in some developing economies. However, this can be addressed by enacting laws that favor vulnerable populations such as the one covered in this review by providing them with full or subsidized Internet coverage.

#### 3.4.2. Adoption of Non-Professional Staff into the Palliative Care Program

**Figure 3:**
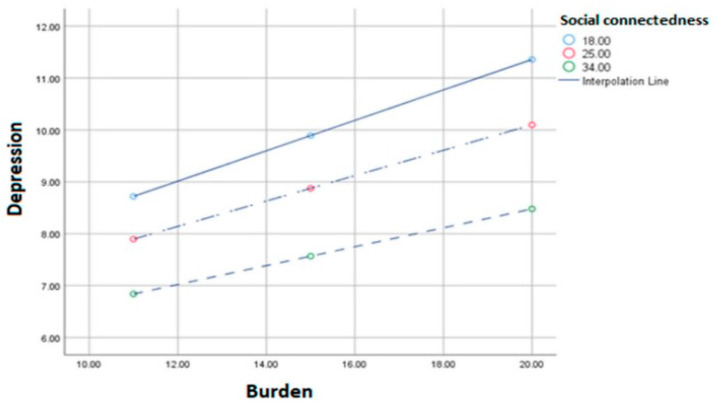
The correlation between the burden of caregiving and depression among caregivers appears to be a growing challenge in palliative care [117].

Cancer specialists are becoming more aware of the value of supportive cancer care. Due to this awareness, there is enthusiasm for creating team-based approaches to regularly provide these services to patients after receiving a cancer diagnosis [115]. Nevertheless, a lot of these team-based strategies rely on qualified individuals to handle advanced care planning and symptom management, including nurses and/or advanced practice practitioners [116]. The demand for the care that cancer patients need, however, is much higher than the number of specialists who can meet it. Patients and caregivers have acknowledged that non-professional staff could also provide supportive cancer care more reliably to close this gap [114]. The strain of caregiving has been positively correlated with signs of anxiety and depression. However, social connectedness appears to operate as a buffer against the negative effects of caregiving, supplying more psychological resources to deal with the stress involved and lowering depression [117]. It is necessary to research methods for boosting caregiver engagement with social networks in ways that increase their sense of social connectivity (Figure 3).

#### 3.4.3. Adoption of Professional Life Coaches and Family Members into the Palliative Care Program

People with cancer are frequently given essential care from family and friends, including practical, physical, emotional, and financial assistance. These people, who are sometimes referred to as informal caregivers in the research literature, are anticipated to take on care that is typically provided by trained clinicians as the provision of supportive care increasingly shifts from the formal health system to the home. These frequently unexpected and difficult caring tasks may have a negative influence on the health and well-being of the caregivers [117]. Giving care to someone with cancer has been regarded as stressful, demanding, and burdensome, even while beneficial results have been noted, such as an enhanced relationship with the care receiver. The term “caregiver burden” refers to the stress that caregivers feel on an emotional, social, physical, financial, and/or spiritual level [118,119]. In addition, our findings acknowledge the need for knowledgeable life coaches in cancer to be facilitated with online platforms in order to effectively coach outpatients where physical distance is an inevitable challenge. The formal oncology systems provide minimal guidance to family members who are responsible for caring for their loved ones with cancer [120]. Few locations connected to cancer treatment facilities exist for families where they can learn how to care for their loved ones during and after treatment or receive information in response to issues that they or their patients confront. A growing number of unfiltered online resources have arisen because of this absence [114]. For these reasons, an extension should also be made focusing on coaching close or immediate family members who oftentimes are left to care for their loved ones in the absence of professional caregivers. In so doing, both local and international life coaches can be contracted to improve the QoL among CRC outpatients and their immediate caregivers. The creation and expansion of an online platform will certainly foster knowledge sharing among experts too and thus improve the quality of palliative care given. As suggested by other authors [114,115], we also place a strong emphasis on examining whether a team-based strategy involving non-professional workers can guarantee the delivery of palliative care services. A Health Coach Support study could close a significant gap in supportive cancer care, and the outcome will certainly spur a better understanding of how to provide cancer patients with a more effective care with an easier functional approach.

### 3.5. Improvement of QoL Outcomes through the Integration of Formal and Informal Palliative Care Systems

It is puzzling that oncology systems have not actively tried to incorporate patients and their families as partners in managing treatments and side effects to obtain optimal patient outcomes given the shifts in the site of treatment from hospitals to outpatient settings and into the home. According to reports [120], as the American population ages, there is a rising demand for cancer treatment services. This has increased the strain on a system that is already understaffed, implementing additional services less likely. Regrettably, there are not any incentives in most of the world’s healthcare systems for the formal system to offer services that go beyond oncology clinics and deliver a more all-encompassing approach to care. It has been proposed that switching from a fee-for-service to a value-based model may encourage the formal care system to think about how informal caregivers (family members) could work with cancer specialists to reduce wasteful spending and enhance results for each treatment program [114,120,121].

Changes in family roles, career shifts, financial and emotional hardship, and changing household routines are just a few of the difficulties that caregivers confront [122]. It has been observed that caregivers and their patients eventually share psychological experiences, particularly with patients at a later stage of their disease [123]. It is important to note that not all family members’ attitudes toward helping a loved one with cancer are unfavorable. There have been reports of favorable reactions to caring, and both positive and negative reactions can occur simultaneously [124]. For many patient–caregiver dyads, positive changes are a shared experience, and getting information from both patients and caregivers about these changes may help clinicians understand more clearly. In the case of advanced CRC, interventions may build on positive developments to encourage a more meaningful QoL [124]. To enhance the science of palliative care in low-resource settings, rigorous experimental investigations and increased quantification of multidimensional palliative care components are required [125].

To obtain the best CRC outpatient outcomes, formal systems such as oncology systems must be more proactive in integrating patients and their loved ones as partners in managing treatments and associated complications. Professionals must be able to assess the patient and the caregiver to ascertain the tasks that each can handle and then ensure that each has the knowledge and tools necessary to complete those duties. Education could be conducted via online media and interaction through health apps that have been expanded to include caregivers, as discussed earlier in this paper.

## 4. Conclusions

The first step in effective pain management is an accurate pain assessment. Pain from CRC remains a feared and unpleasant side effect of both cancer and cancer treatment. It is a prevalent and debilitating cancer symptom that can disrupt outpatients’ lives more than the illness itself. Untreated or improperly treated pain can have serious repercussions, affecting essential components of QoL, such as physical well-being, psychological functioning, and interpersonal relationships. Physical pain management, symptom control, information exchange, advance care planning, spiritual and emotional support, and care coordination are the main issues associated with pain and relevant to palliative care. An accurate examination of disruptive pain disorders is necessary to alleviate them and their effects among patients under palliative care to enhance patient health, longevity, responsiveness to therapy, and decrease comorbidity complications. Physical activity has been demonstrated to help lessen pain and its associated factors, like anxiety, sleep disorder, sadness, fatigue, and lymphedema, among cancer survivors receiving palliative care. Outpatient pain management could benefit tremendously from improved, validated, and culturally translated approaches and algorithms, as proposed in our updated study. Physical activities, music and massage therapies, the use of pain assessment tools, remote outpatient education and monitoring, chemotherapeutic pain reduction strategies, and bridging social isolation gaps are essential in improving QoL among CRC outpatients. Finally, we recommend and place a strong emphasis on the adoption of online support platforms and programs (coaching and training) and the integration of formal and informal palliative care systems for maximum QoL benefits among CRC outpatients receiving palliative care.

## Figures and Tables

**Table 1:** A simple validated pain assessment tool that can be deployed for CRC outpatient pain measurements [33].

Tool	Letter and Its Representation	Descriptive Questions
OLDCART	O—onset	- When did the pain start?
	L—location	- Where is the pain? Is there more than one location?
	D—duration	- How often does the pain occur? Is it constant or intermittent? How long does the pain last?
	C—characteristics	- How does the pain feel (intensity)? What words would you use to describe the pain?
	A—aggravating factors	- What makes the pain worse?
	R—relieving factors	- What makes the pain better?
	T—treatment	- What treatments have you tried to control the pain? How are they working? How do they affect the pain intensity?
PQRST	P—provocation/palliation	- What causes or relieves it?
	Q—quality	- What does it feel like?
	R—region/radiation	- Where is the pain? Does the pain radiate?
	S—severity	- How severe is the pain on a 0-to-10 scale?
	T—riming	- Constant or intermittent?
WILDA	W—words	- The words used to describe your pain.
	I—intensity	- On a 0–10 scale, what is your pain now, at rest, on movement, worst pain possible in past 24 h? What is your comfort/function goal?
	L—location	- Where is your pain?
	D—duration	- Is your pain always there, or does it come and go? Do you have both types of pain?
	A—aggravating/alleviating factors	- What makes your pain worse or better?
MOPAT	M—multidimensional	This tool can be used to assess pain in noncommunicative CRC patients in the critical care environment. The MOPAT is distinctive in that it can be deployed over time and in different settings.
	O—objective	
	P—pain	
	A—assessment	
	T—tool	

## Data Availability

The datasets generated during and/or analyzed during the current study are available from the corresponding author upon reasonable request.
